# Superior mesenteric artery margin in pancreaticoduodenectomy for pancreatic adenocarcinoma

**DOI:** 10.18632/oncotarget.13950

**Published:** 2016-12-15

**Authors:** Dao-ning Liu, Ang Lv, Zhi-hua Tian, Xiu-yun Tian, Xiao-ya Guan, Bin Dong, Min Zhao, Chun-yi Hao

**Affiliations:** ^1^ Department of Hepato-Pancreato-Biliary Surgery, Peking University Cancer Hospital and Institute, Beijing, People's Republic of China; ^2^ Key Laboratory of Carcinogenesis and Translational Research, Ministry of Education, Beijing, People's Republic of China; ^3^ Department of Pathology, Peking University Cancer Hospital and Institute, Beijing, People's Republic of China

**Keywords:** pancreatic cancer, tumor budding, superior mesenteric artery margin, R0 surgery, epithelial–mesenchymal transition

## Abstract

The aim of this study is trying to describe more details of superior mesenteric artery margin in pancreaticoduodenectomy for pancreatic ductal adenocarcinoma, to evaluate biological and prognostic implications of tumor budding in this margin, and to provide more evidence for evaluation of R0 surgery in pancreaticoduodenectomy. 46 patients in 5-years period are included in this study. Immunochemistry and immunofluorescence are used to analyze tumor budding and epithelial–mesenchymal transition. Superior mesenteric artery margin might be described from four aspects including location, gross appearance, microscopic appearance and tumor budding. We find that 1mm rule for R1 surgery is more appropriate to predict prognosis (*P* = 0.009) than 0mm rule (*P* = 0.141). Expression of cytokeratin in tumor budding is significantly lower than primary tumor (*P* = 0.001), and it suggests that tumor budding may participate the procedure of epithelial–mesenchymal transition. High-grade tumor budding and decreasing cytokeratin of tumor budding correlate with distant metastasis and has negative influence on prognosis. So superior mesenteric artery margin might be not only an area that tumor cells may invade, but also a pathway for distant metastasis. It is necessary to evaluate superior mesenteric artery margin in pancreaticoduodenectomy for pancreatic cancer.

## INTRODUCTION

Pancreatic ductal adenocarcinoma (PDAC) is one of the most lethal malignancies. Five-year survival rate is extremely low. Tumor resection is the only effective treatment and achieving R0 resection is critical for long-term survival [1, 2], however, long-term survival after pancreaticoduodenectomy cannot be reliably predicted [3]. Evaluation of superior mesenteric artery (SMA) margin is one of the key problems.

Epithelial–mesenchymal transition (EMT) of adherent epithelial cells to migratory mesenchymal status has been implicated in tumor metastasis in preclinical models [4]. Relationship between EMT and metastasis is now a controversial problem [5–10].

Tumor budding is a type of infiltrative growth pattern consisting of isolated tumor cells or small cell clusters (< 5 cells) located at the invasive front of some types of carcinomas [11, 12]. It is suggested that tumor budding might represent local dissemination of cancer cells into surrounding tissue, and correlate with EMT [3, 13, 14]. Tumor budding has been proved to be an independent prognostic factor of PDAC, however, these studies are refined intra-tumor and retrospective [11, 12]. It is hard to confirm that the invasive front (surgical margin) where tumor budding and EMT really exist.

The SMA margin was described as the most important margin by the NCCN guidelines. It was described as the soft tissue directly adjacent to the proximal 3–4 cm of SMA [15]. In our study, we are trying to describe more details of SMA margin, which might be helpful for surgeons to understand the SMA margin comprehensively.

## RESULTS

According to the inclusion and exclusion criteria, in total 46 patients are included in the current study (Table 1). The average of overall survival (OS) is 26.20 months, disease free survival (DFS) is a 17.39 months, and five-year survival rate is 26.5%.

**Table 1:** Clinical and pathological characteristics in the total patient cohort, tumor budding group, and R1 (1 mm) group

Parameters		*n*	Low grade	High grade	*P*-value	< 1 mm	> 1 mm	*P*-value
**Age (y)**		60.87 ± 11.27	60.90 ± 11.22	60.82 ± 11.70	0.983	64.71 ± 10.89	58.62 ± 11.05	0.077
**Operative time (min)**		478.98 ± 71.46	465.93 ± 65.14	501.24 ± 78.11	0.106	502.41 ± 79.57	465.24 ± 63.71	0.089
**Blood loss (ml)**		611.96 ± 342.41	644.83 ± 352.12	555.89 ± 327.82	0.401	682.35 ± 300.49	570.69 ± 363.40	0.291
**Gender**					0.858			0.650
	**Male**	29 (63%)	18 (62.1%)	11 (64.7%)		10 (58.8%)	19 (65.5%)	
	**Female**	17 (37%)	11 (37.9%)	6 (35.3%)		7 (41.2%)	10 (34.5%)	
**Tumor size (cm)**					0.151			0.762
	**> 3 cm**	34 (73.9%)	24 (82.8%)	10 (58.8%)		13(76.5%)	21 (72.4%)	
	**< 3 cm**	12 (26.1%)	5 (17.2%)	7 (41.2%)		4 (23.5%)	8 (27.6%)	
**Histological grade**					0.783			0.930
	**Low**	20 (43.5%)	13 (44.8%)	7 (41.2%)		7 (41.2%)	13 (44.8%)	
	**Middle**	20 (43.5%)	13 (44.8%)	7 (41.2%)		8 (47.1%)	12 (41.4%)	
	**High**	6 (13%)	3 (10.3%)	3 (17.6%)		2 (11.8%)	4 (13.8%)	
**T stage**					0.478			0.798
	**T1**	0	0	0		0	0	
	**T2**	6 (13%)	3 (10.3%)	3 (17.6%)		3 (17.6%)	3 (10.3%)	
	**T3**	40 (87%)	26 (89.7%)	14 (82.4%)		14(82.4%)	26 (89.3%)	
	**T4**	0	0	0		0	0	
**N stage**					0.858			0.650
	**N0**	17 (37%)	11 (37.9%)	6 (35.3%)		7 (41.2%)	10 (34.5%)	
	**N1**	29 (63%)	18 (62.1%)	11 (64.7%)		10 (58.8%)	19 (65.5%)	
**Complication (Dindo–Clavien Classification)**					0.631			0.439
	**I**	10 (21.7%)	4 (13.8%)	6 (35.3%)		5 (29.4%)	5 (17.2%)	
	**II**	17 (37%)	12 (41.4%)	5 (29.4%)		5 (29.4%)	12 (41.4%)	
	**IIIa**	14 (30.4%)	10 (34.5%)	4 (23.5%)		4 (23.5%)	10 (34.5%)	
	**IIIb**	5 (10.9%)	3 (10.3%)	2 (11.8%)		3(17.6%)	2 (6.9%)	
	**IV**	0	0	0		0	0	
**Pancreatic fistula (ISGPF)**					0.803			0.631
	**None**	37 (80.4%)	23 (79.3%)	14 (82.4%)		14 (82.4%)	23 (79.3%)	
	**A**	5 (10.9%)	4 (13.8%)	1 (5.9%)		1 (5.9%)	4 (13.8%)	
	**B**	4 (8.7%)	2 (6.9%)	2(11.8%)		2 (11.8%)	2 (6.9%)	
	**C**	0	0	0		0	0	
**Adjuvant therapy**		20 (43.5%)	12 (41.4%)	8 (47.1%)	0.708	7 (41.2%)	13 (44.8%)	0.809
**SMV/PV resection**		30 (65.2%)	19 (65.5%)	11 (64.7%)	0.956	14 (82.4%)	16 (55.2%)	0.062
**Positive PV/PVG margin**		9 (19.6%)	5 (17.2%)	4 (23.5%)	0.604	6 (35.3%)	3 (10.3%)	**0.04**
**Perineural invasion**		31 (67.4%)	20 (69%)	11 (64.7%)	0.766	11 (64.7%)	20 (69%)	0.766
**Lymph node ratio**					0.106			0.478
	**> 0.2**	6 (13%)	2 (6.9%)	4 (23.5%)		3 (17.6%)	3 (10.3%)	
	**< 0.2**	40 (87%)	27 (93.1%)	13 (76.5%)		14 (82.4%)	26 (89.7%)	
**Lymphovascular invasion**		17 (37%)	8 (27.6%)	9 (52.9%)	0.085	8 (47.1%)	9 (31%)	0.227
**Reoperation**		7 (15.2%)	3 (10.3%)	4 (23.5%)	0.229	4 (23.5%)	3 (10.3%)	0.229
**Length of stay (days)**		42.54 ± 23.74	44.24 ± 26.57	39.65 ± 18.32	0.532	45.12 ± 31.02	41.03 ± 18.70	0.579
**Total pancreaticoduodenectomy**		11 (23.9%)	6 (20.7%)	5 (29.4%)	0.402	5 (29.4%)	6 (20.7%)	0.564
**Resection of other organs**		8 (17.4%)	5 (17.2%)	3 (17.6%)	0.402	4 (23.5%)	4 (13.8%)	0.564
**Distant metastasis**		25 (54.3%)	16 (64%)	9 (36%)	0.002	12 (60%)	13 (40%)	0.09
**Local recurrence**		20 (43.5%)	11 (55%)	9 (45%)	0.167	10 (50%)	10 (50%)	0.108

For immunochemistry, tumor cells are not found in 7 patients’ specimen because the primary tumor stays far from the SMA margin. Low-grade tumor budding is observed in 19 patients. These 26 (56.5%) patients have been regarded as low-grade group. Another 20 (43.5%) patients who possess high-grade tumor budding have been regarded as high-grade group. Patients in high-grade group have worse OS and DFS using univariate statistic, *P* = 0.006, *P* = 0.000, respectively. In Cox multivariate analysis, high-grade tumor budding is also an independent prognostic factor for OS and DFS (*P* = 0.01, *P* = 0.001, respectively). Without considering the 7 patients who do not have tumor cells in the specimen, high-grade group still shows worse OS and DFS (*P* = 0.04, *P* = 0.003, respectively).

**Figure 1:**
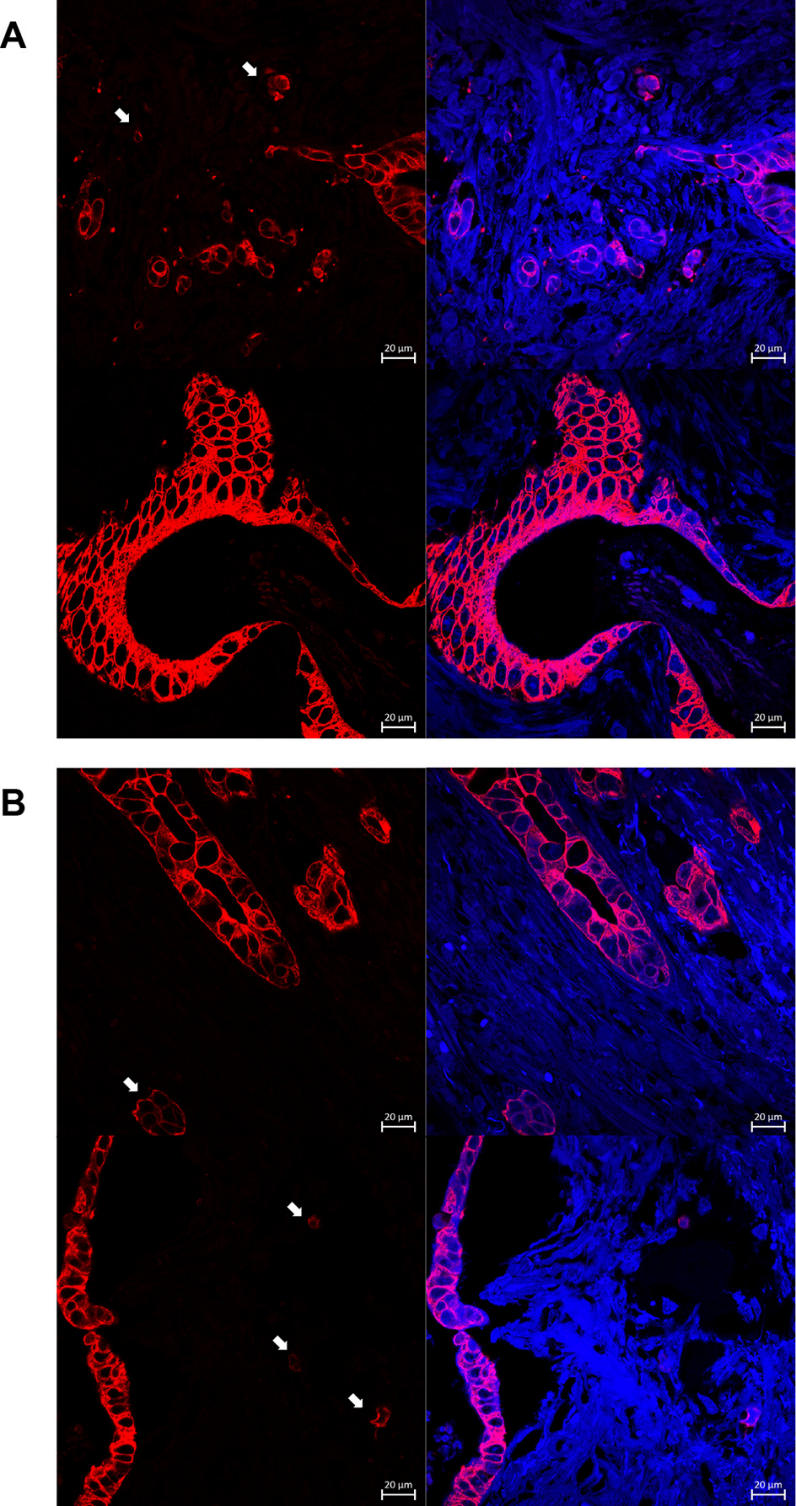
These pictures show results of immunofluorescence Figure (**A**) shows the invasive front and primary of the tumor. Figure (**B**) shows the tumor budding and their decreasing fluorescence intensity value.

For immunofluorescence, fluorescence intensity value of primary tumor is significantly higher than tumor budding in 33 patients. They will be regarded as decreasing cytokeratin group. On the contrary, other patients will be regarded as not (Figure 1). Patients possessing decreasing cytokeratin also suggest worse OS and DFS, *P* = 0.041, *P* = 0.002, respectively.

Furthermore, it is noteworthy that the presence of high-grade tumor budding and decreasing cytokeratin correlate with distant metastasis (*P* = 0.002, *P* = 0.000, respectively). For all patients, the intensity value of tumor budding (54.34 ± 23.24) is significantly lower than the primary tumor (161.16 ± 44.14), *P* = 0.001.

**Figure 2:**
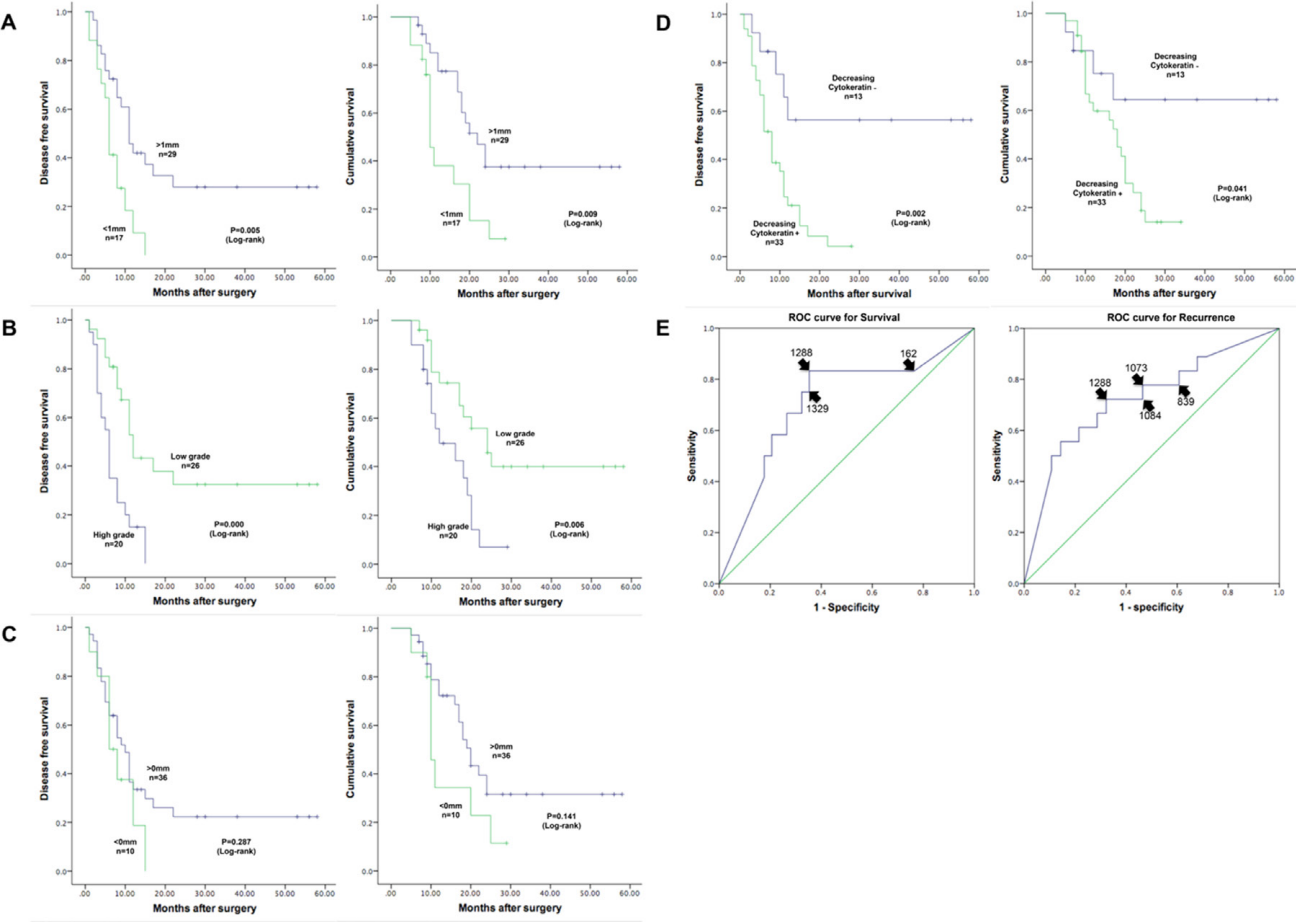
Figure (**A**) shows that R1 surgery (1 mm rule) has adverse effect on OS and DFS using Kaplan-Meier survival curves. (**C**) shows that 0 mm rule does not. (**B**) shows that high grade tumor budding affects OS and DFS. (**D**) shows that decreasing cytokeratin affects OS and DFS. E shows the ROC curve for different R1 rule to evaluate survival and recurrence.

0 mm rule for R1 surgery shows no effect on OS and DFS (*P* = 0.141, *P* = 0.287, respectively). However, it correlates with local recurrence (*P* = 0.041). Furthermore, 1 mm rule has advert effect on OS and DFS (*P* = 0.009, *P* = 0.005, respectively), but it shows no correlation with local recurrence (*P* = 0.108). Receiver operating characteristic (ROC) curve suggests that 1mm rule is appropriate to evaluate survival or recurrence of PDAC. (Figure 2).

**Figure 3:**
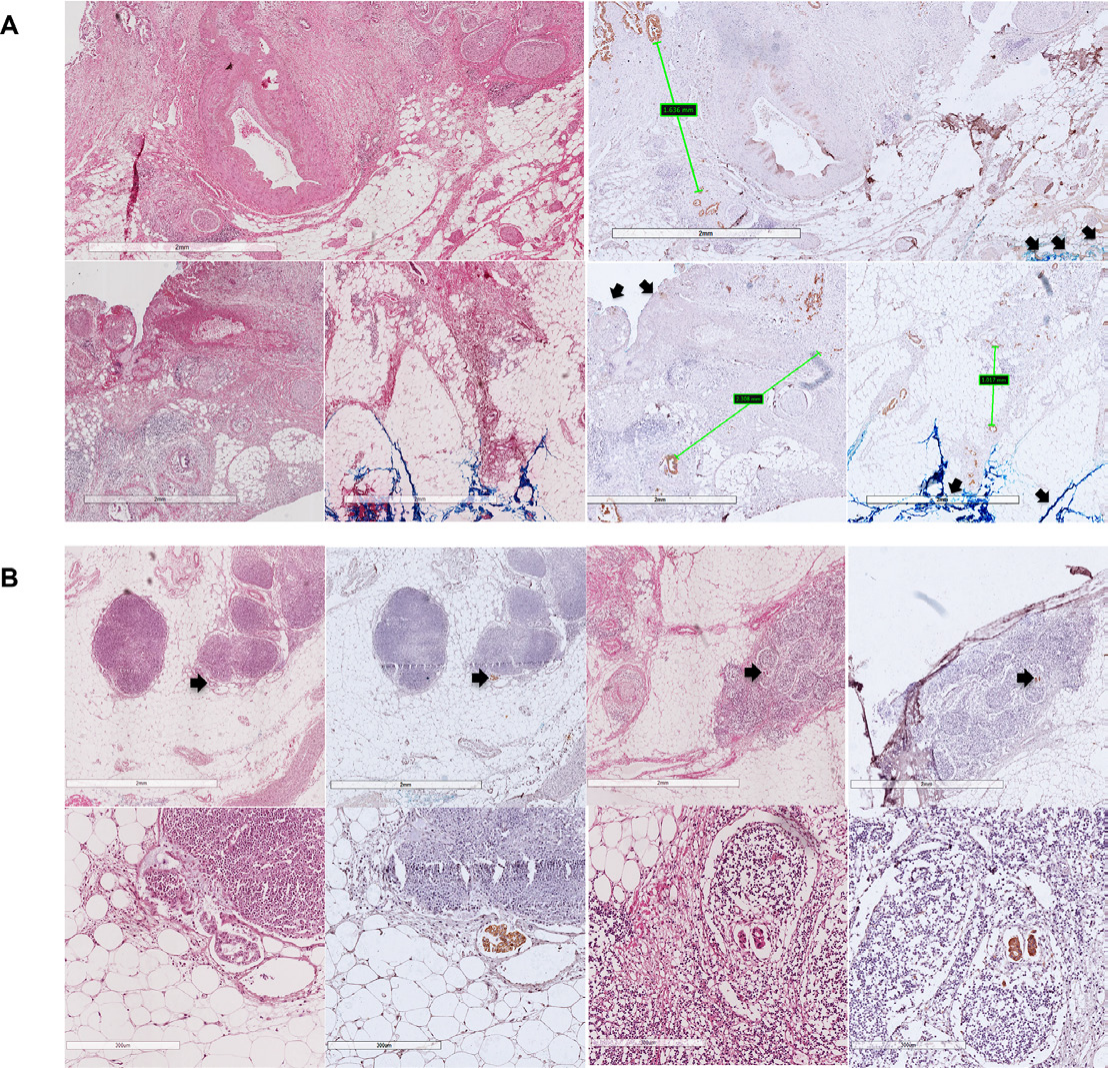
These pictures show how dispersed the distribution of PDAC could be Figure (**A**) shows the maximum distance between tumor cells, and figure (**B**) show lymphovascular invasion that have been observed. Arrows show the SMA margin.

In 5 cases, the maximum distance between neighboring tumor cells is greater than 1mm. All of these patients experienced recurrence and death after the operation, but we did not get a positive statistical result. The maximum distance does not correlate with high-grade tumor budding or decreasing cytokeratin (*P* = 0.092, *P* = 0.077, respectively). Another phenomenon that some tumor cells invaded far away from the primary tumor has been observed in 3 cases, and these tumor cells are evaluated as lymphovascular invasion (Figure 3).

In our study, we are trying to describe more details of SMA margin, and this description will help us to perform the operation. The four parts of the description are listed below.

### Location

The name of SMA margin is chaotic in different studies, it was named as SMA margin, retroperitoneal margin, posterior margin, uncinate margin or mesenteric margin before. According to these names, we would relate it with margin locates at dorsal to the pancreatic head, adjacent to superior mesenteric artery, and might contain the mesopancreas. The definition of mesopancreas differs in different studies [16–21]. However, we can find some similarities between SMA margin and mesopancreas. They both locate at dorsal to the pancreas, along SMA to aorta [19]. Some studies also suggest that the skeleton of SMA and specific part of aorta can maximize the SMA margin [22, 23]. These conclusions which include mesopancreas, region of lymph node dissection and skeleton of SMA might describe the same problem from different aspects [24]. According to these descriptions, we define mesopancreas as a soft connective tissue along SMA to the right anterior surface of aorta and confined in the pancreatic area. We integrate this mesopancreas into our definition SMA margin, and use this definition to help us perform the meso-pancreatoduodenum excision procedure.

### Gross appearance

Some scholars put forward the definitions of transection margin and mobilization margin. They drew a conclusion that positive mobilization margin alone did not influence the survival of PDAC patients [25]. Based on these definitions, we believe that the SMA margin is a coarse transection margin that has been resected from SMA, but not a smooth mobilization margin that mobilized through a natural space, which locates at dorsal to retropancreatic fusion fascia. Normally when we get a specimen, it is easy to find the smooth retropancreatic fusion fascia (posterior margin) at the dorsal side. The end of this smooth fascia is just the beginning of SMA margin, then SMA margin continues until portal vein/portal vein groove (PV/PVG) emerges.

### Microscopic appearance

Retropancreatic fusion fascia is an important definition. From the description of Baik Hwan Cho's study, it is a critical anatomical landmark during retropancreatic mobilization of pancreatic head and duodenum (the Kocher maneuver). The pancreatic parenchyma, extrapancreatic nerve plexuses, SMA and PV/SMV are wrapped within the fusion fascia and exist in the same space in adults [26]. To protect SMA, pancreaticoduodenectomy will break retropancreatic fusion fascia, and the broken place is the exact position of SMA margin. HIROHISA KITAGAWA's study also proved that retropancreatic fusion fascia acts as a barrier against infiltration by PDAC [27]. In conclusion, pancreaticoduodenectomy will break retropancreatic fusion fascia, which acts as a barrier, and expose the dangerous place where the tumor cells always invade. These descriptions may explain the result of some former studies that PDAC patients benefit from routine resection of SMV/PV and total meso-pancreatoduodenum excision, as these procedures provide a smaller broken area of retropancreatic fusion fascia than normal procedures [28–30].

### Tumor budding and EMT

As mentioned above, tumor budding has been proved to be an independent prognostic factor and might correlate with EMT. In our view, it is very important and meaningful to observe the behavior of tumor buddings at surgical margin. As the theory mentioned in Caroline S. Verbeke‘s studies that tumor growth in PDAC is more dispersed than in rectal cancer [31, 32]. Based on the characteristic of EMT and tumor budding, we suppose that this disperse behavior may be caused by tumor budding and EMT, and SMA margin might be a pathway for metastasis.

## DISCUSSION

For surgical oncology, surgical margin is always a vital problem. As circumferential resection margin and total mesorectal excision have become the consensus for rectal cancer [33, 34], alike definitions are introduced into pancreaticoduodenectomy. Besides traditional margins including bile duct margin, pancreatic neck margin, proximal and distal enteric margin, new margins have been studied. In these studies, anterior and posterior margin are less important because the studies of mobilization margin and inhibition by retropancreatic fusion fascia [25, 27]. As PV/PVG margin can be solved by routine resection of SMV/PV, we focus on the SMA margin. As a surgical margin that can predict prognosis and judge surgery, SMA margin should possess two important factors. One is that, like pancreatic neck margin breaking pancreas, SMA margin breaks retropancreatic fusion fascia. It is produced by breaking some normal tissue or organs (transection, sharp dissection), but not in a way of getting through (mobilization, blunt dissection). The other important factor is that production of the SMA margin is like opening the “gate” for cancer cells to invasion and especially metastasis. To investigate metastasis, EMT and tumor budding [3, 9, 10, 13, 14, 35, 36], we designed this study.

In terms of the invasion aspect, we focus on R1 surgery, as there is still a lack of consensus of R1 rule. In the past, represented by AJCC [37] 0 mm rule is applied in North America. However, in Europe, represented by The Royal College of Pathologists [38] 1mm rule is preferred. In our study, we drew the conclusion that 0 mm rule may be a better judgement for tumor residue but not enough for prognosis. ROC and Kaplan–Meier curve both support 1mm rule. We observed that some tumor cells could be very dispersed (> 1 mm) and invaded far away from primary tumor. Although these phenomena did not show effect on prognosis, they did prove the hypothesis posed by Verbeke [31]. These negative results for prognosis may happen when primary tumor stays far away from SMA margin. On the other hand, We did not find the relationship between R1 status of PV/PVG margin and prognosis, we are convinced that this is because our aggressive choice of PV/SMV resection (65.2%). This aggressive operation may weaken the influence of positive portal vein margin.

From the metastasis aspect, tumor budding and EMT are another interesting part in our study [13, 39]. EMT gives rise to the dissemination of single cancer cells from primary tumor, which is similar with the definition of tumor budding [40]. Several studies proved the relationship between E-Cadherin, β-catenin, snail, ZEB1, ZEB2 expression and tumor budding using immunochemistry, and drew the conclusion of EMT induction at the tumor–host interface, but more evidence are required [3, 41]. In our study, we evaluated tumor budding at SMA margin and proved two hypotheses. For one thing, decreasing expression of cytokeratin supports that tumor budding participates EMT procedure. For another, we proved that tumor budding and EMT does correlate with metastasis, as EMT events in surgical margin are the most possible place to cause distant metastasis. In conclusion, SMA margin is a pathway for metastasis.

Chemoresistance for PDAC is another problem that lacks consensus. The main reason of this problem is the low response rate for chemotherapy [42–46]. In our study, every patients accepted adjuvant chemotherapy if physical condition permits, and 20 patients (43.5%) in total accept chemotherapy (gemcitabine + S1) after the operation. We do not find adjuvant chemotherapy have positive influence on DFS (*P* = 0.114). As EMT is responsible for chemoresistance, we suppose that tumor budding may possess the same feature. So we exclude high-grade patients, the adjuvant chemotherapy still did not affect DFS (*P* = 0.066), but its effect is more significant. This conclusion may help us to select suitable patients for adjuvant chemotherapy, and make a more precise prediction of prognosis.

In the past years, we have found that SMA margin is a very interesting and meaningful area for PDAC. This area involves R1 surgery, prognosis, resectability, neoadjuvant and adjuvant chemotherapy of PDAC. Because of small sample size in our study, many phenomena, like relationship between dispersiveness of PDAC and EMT, could not be studied clearly. In the next step of our study, we want to investigate the mechanism of EMT in this margin and its effect on prognosis, as there are some disadvantageous evidence have been reported recently at animal experiments [9, 10]. As tumor budding may form distant metastasis, possible correlation between tumor budding, circulating tumor cell and cancer stem cell is another interesting hypothesis waiting for us to study. If any part of tumor budding can be confirmed as cancer stem cell, it will be an ideal target for studying chemoresistance and cancer stem cell-targeting therapy.

## MATERIALS AND METHODS

### Patients

Patients are included using the inclusion and exclusion criteria. All patients accept pancreaticoduodenectomy or total pancreaticoduodenectomy, combined with or without resection of SMV/PV or other organs in Department of Hepato-Pancreato-Biliary Surgery, Peking University Cancer Hospital during a 5-year period (April, 2011 to September, 2015). Ethical approval and written informed consent have been obtained. Clinical, pathological and prognostic information have been collected.

### Inclusion and exclusion criteria

(1) Patients whose preoperative diagnosis and postoperative pathology are both PDAC will be included, and they receive PD or total PD.

(2) Patients do not receive any anticancer treatment before the operation.

(3) All patients accept R0/R1 resection, and those who accept R2 resection will be excluded.

(4) No distant metastasis is found before/during the operation.

(5) All patients have signed the informed consent and agreed to participate this study.

(6) Those patients who die of perioperative complications will be excluded.

(7) Expect for gemcitabine + S1, patients who accept other adjuvant chemotherapy will be excluded.

### Surgical technique

**Figure 4:**
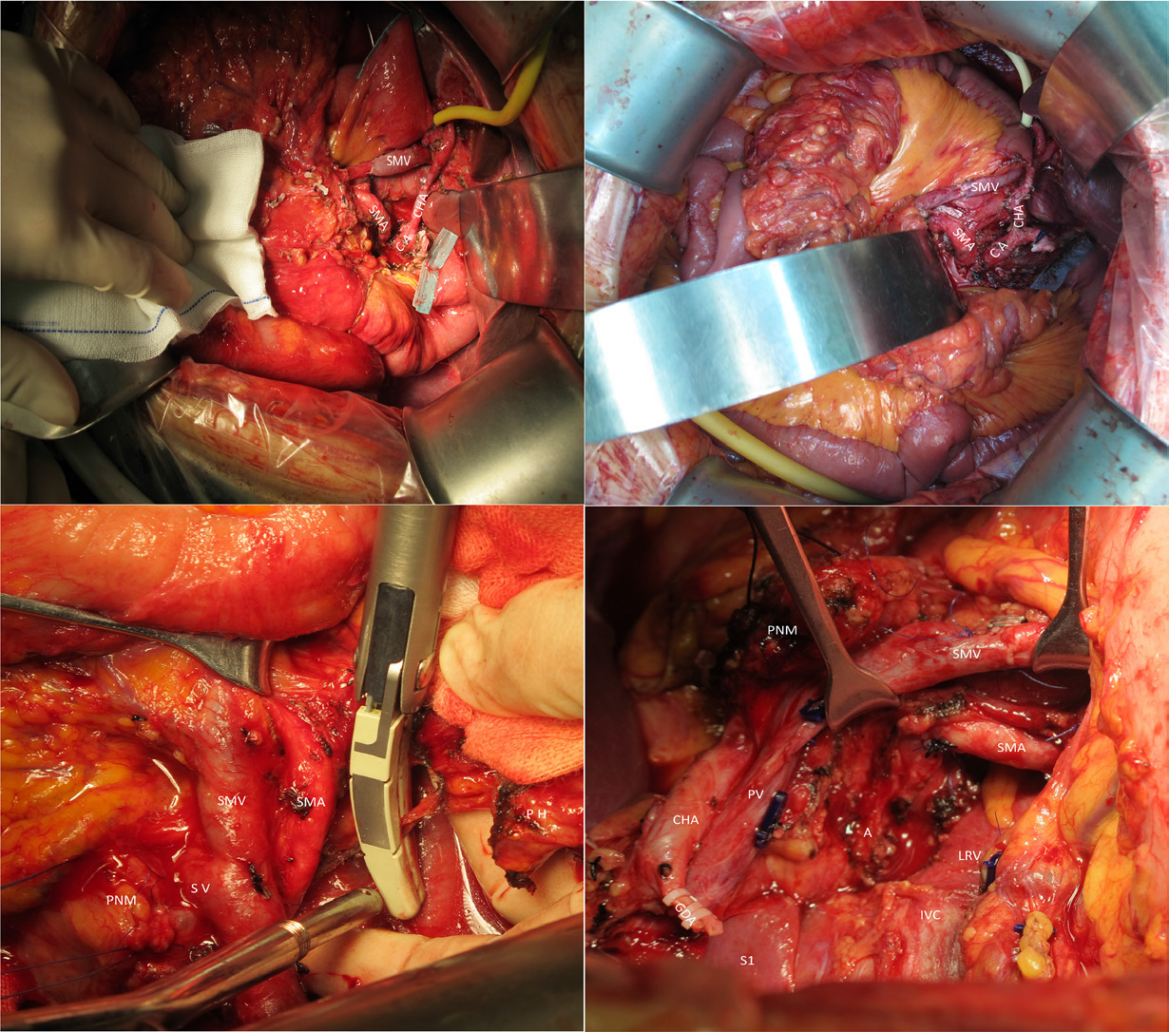
These pictures show the range of our surgery to get a integrate SMA margin SMA superior mesenteric artery, SMV superior mesenteric vein, CA celiac axis, CHA common hepatic artery, PNM pancreatic neck margin, A Aorta, LRV left renal vein, IVC inferior vena cava, S1 segment 1 of liver, GDA gastroduodenal artery, PV portal vein.

An upper midline incision is usually made. The greater omentum is firstly separated from transverse colon to identify the anterior surface of the pancreas. Then the colon is fully mobilized to expose the infrapancreatic SMV. Kocher maneuver is performed to expose the place where left renal vein contributes into the inferior vena cava (IVC), as it will help confirm the position of SMA. The lesser omentum is divided along hepatic artery to the origin of celiac axis (CA), and gastroduodenal artery (GDA) will be ligated. After removing the gallbladder, common hepatic duct, stomach, and jejunum will be dissected. Pancreatic neck is dissected in front of SMV. We emphasize the method of how to dissect the pancreatic uncinate process, where the SMA margin is. To get an assessable SMA margin, electrocautery and ligation will be the first choice to dissect pancreatic uncinate process, but not using a load stapler. A meso-pancreatoduodenum excision procedure is made to ensure en-bloc resection of the mesopancreas with the specimen. The skeleton of SMA and right anterior part of aorta (from SMA to CA) is operated to maximize the margin. During this procedure, the first jejuna artery (FJA) is carefully conserved and the inferior pancreaticoduodenal artery (IPDA) is ligated. If tumor infiltration is found, the SMV will be partially resected and reconstructed either by end-to-end anastomosis or insertion of a venous graft. (Figure 4).

### Tissue processing

**Figure 5:**
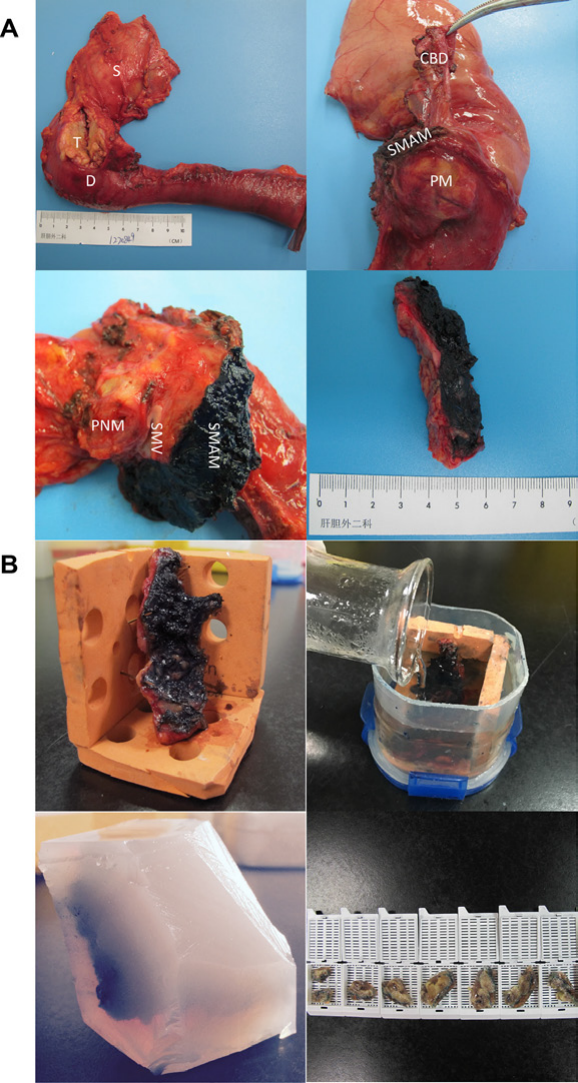
These pictures show the process of how we get and manage the SMA margin S stomach, T tumor, D duodenum, CBD common bile duct, SMAM superior mesenteric artery margin, SMV superior mesenteric vein, PM posterior margin, PNM pancreatic neck margin.

According to the definition of SMA margin, the margin was found and marked using India ink, right after the specimen was resected off. Then the margin was departed from the specimen. The thickness of the tissue is always more than 1cm, because we are trying to get a margin that contains normal pancreas and part of the primary tumor. Furthermore, we design a unique approach to prevent the margin from shrinking. As is shown in Figure 5, after we used the orange model to fix the margin in natural and flat status, the specimen was fixed by formalin for about 2 hours. Then the whole model was embedded by 3% agarose gel. In this way, we can get a perfect margin just like it was in the body and it will not affect immunochemistry. After completing, the specimen is paraffin-embedded. Each fragment is cut into 4 μm and stained for HE, then the distance between tumor cells and SMA margin are measured for every section. The fragment that possesses the closest distance is used to describe R status. Except for the SMA margin, PV/PVG margin and traditional margins are evaluated by pathology department. The posterior and anterior margin are not included in our study, because former studies have already proved their weak affect on prognosis [25, 27].

### Immunohistochemistry and immunofluorescence

Immunochemistry has been performed using Anti-pan Cytokeratin antibody [AE1+AE3] (1:200, ab961, Abcam, Cambridge, UK) to highlight areas of tumor budding. Without 9 patients who did not possess primary tumor in SMA margin, sections of 37 patients were adopted for immunofluorescence using Anti-pan Cytokeratin antibody [PCK-26] (1:500, ab401, Abcam, Cambridge, UK). Negative controls have been established to eliminate nonspecific fluorescence. Fluorescence intensity value of primary tumor (> 200 cells) and all tumor budding has been measured by laser confocal scanning microscope (LSM 780, ZEISS, Germany) and ZEISS ZEN software. To guarantee comparability, photograph was captured in certain condition using Plan-Apochromat 63 × /1.40 Oil DIC M27 (master gain is 800, digital gain is 1.1, digital offset is 0 and laser line attenuator transmission in 9%).

### Assessment of tumor budding

**Figure 6:**
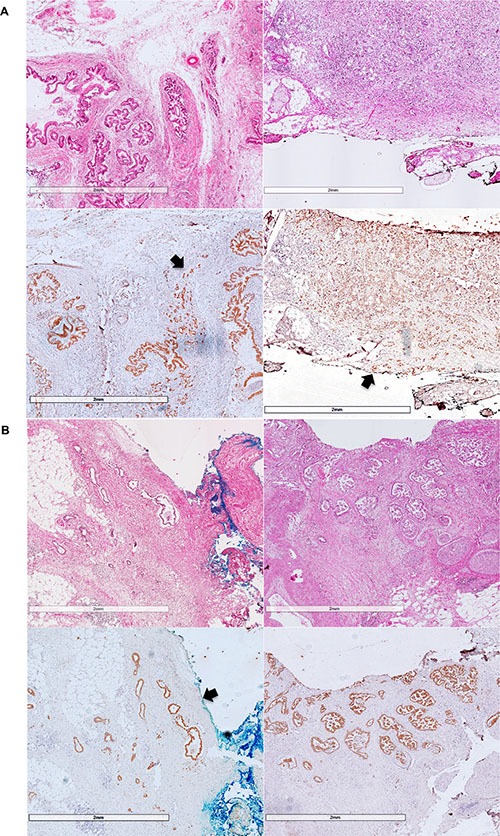
Figure (**A**) shows examples for high grade tumor-budding, and figure (**B**) shows examples for low grade tumor-budding. The arrows show the SMA margin and invasive front of the tumor.

According to the definition of tumor budding, it is de-differentiated single cells or clusters of < 5 cells in central or periphery of the tumor. Tumor budding will be evaluated by HE, immunochemistry and immunofluorescence. Two experienced pathologists selected the highest density of tumor budding for assessment. Tumor budding will be graded as low (≤ 10TB/HPF) versus high (> 10TB/HPF). (Figure 6).

### Dispersiveness of tumor cell

To investigate dispersiveness of infiltrating tumor cells, distance between two nearest neighboring tumor cells has been measured. The maximum distance has been selected to represent the dispersiveness of PDAC, as former study has proved that tumor cells are more dispersed in the tumor periphery than the central part [32]. (Figure 6).

### Statistics

Data collection and statistical analysis were performed with IBM SPSS Version 21 (SPSS Inc, Chicago, IL, USA). Enumeration data were expressed as mean and standard deviation, ranked data by cross-tabulation and percentages, and survival data by Kaplan–Meier method. For statistical analysis, *T* test, McNemar, chi-square test, and log–rank test were employed. Multivariate modeling was performed by binary logistic regression. All tests were performed two-sided at a significance level of *P* = 0.05.

## Abbreviations

PDAC: pancreatic ductal EMT
adenocarcinoma: epithelial–mesenchymal transition
SMA: superior mesenteric artery
OS: overall survival
DFS: disease free survival
ROC: receiver operating characteristic curve
PV/PVG: portal vein/portal vein groove
IVC: inferior vena cava
CA: celiac axis
GDA: gastroduodenal artery
FJA: first jejuna artery
IPDA: inferior pancreaticoduodenal artery

## References

[1] Bilimoria KY, Talamonti MS, Sener SF, Bilimoria MM, Stewart AK, Winchester DP, Ko CY, Bentrem DJ (2008). Effect of Hospital Volume on Margin Status after Pancreaticoduodenectomy for Cancer. Journal of the American College of Surgeons.

[2] Winter JM, Cameron JL, Campbell KA, Arnold MA, Chang DC, Coleman J, Hodgin MB, Sauter PK, Hruban RH, Riall TS, Schulick RD, Choti MA, Lillemoe KD (2006). 1423 pancreaticoduodenectomies for pancreatic cancer: A single-institution experience. J Gastrointest Surg.

[3] Kohler I, Bronsert P, Timme S, Werner M, Brabletz T, Hopt UT, Schilling O, Bausch D, Keck T, Wellner UF (2015). Detailed analysis of epithelial-mesenchymal transition and tumor budding identifies predictors of long-term survival in pancreatic ductal adenocarcinoma. J Gastroenterol Hepatol.

[4] Yu M, Bardia A, Wittner BS, Stott SL, Smas ME, Ting DT, Isakoff SJ, Ciciliano JC, Wells MN, Shah AM, Concannon KF, Donaldson MC, Sequist LV (2013). Circulating breast tumor cells exhibit dynamic changes in epithelial and mesenchymal composition. Science.

[5] Stewart CJ, Little L (2009). Immunophenotypic features of MELF pattern invasion in endometrial adenocarcinoma: evidence for epithelial-mesenchymal transition. Histopathology.

[6] Brabletz T, Jung A, Spaderna S, Hlubek F, Kirchner T (2005). Opinion: Migrating cancer stem cells—An integrated concept of malignant tumour progression. Nature Reviews Cancer.

[7] Brabletz T (2012). To differentiate or not--routes towards metastasis. Nat Rev Cancer.

[8] Jeong H, Ryu YJ, An J, Lee Y, Kim A (2012). Epithelial-mesenchymal transition in breast cancer correlates with high histological grade and triple-negative phenotype. Histopathology.

[9] Zheng X, Carstens JL, Kim J, Scheible M, Kaye J, Sugimoto H, Wu CC, Lebleu VS, Kalluri R (2015). Epithelial-to-mesenchymal transition is dispensable for metastasis but induces chemoresistance in pancreatic cancer. Nature.

[10] Fischer KR, Durrans A, Lee S, Sheng J, Li F, Wong ST, Choi H, El RT, Ryu S, Troeger J (2015). Epithelial-to-mesenchymal transition is not required for lung metastasis but contributes to chemoresistance. Nature.

[11] Kate OC, Li-Chang HH, Kalloger SE, Peixoto RD, Webber DL, Owen DA, Driman DK, Richard K, Stefano S, Scudamore CH (2015). Tumor budding is an independent adverse prognostic factor in pancreatic ductal adenocarcinoma. American Journal of Surgical Pathology.

[12] Karamitopoulou E, Zlobec I, Born D, Kondi-Pafiti A, Lykoudis P, Mellou A, Gennatas K, Gloor B, Lugli A (2013). Tumour budding is a strong and independent prognostic factor in pancreatic cancer. Eur J Cancer.

[13] Karamitopoulou E (2013). Role of epithelial-mesenchymal transition in pancreatic ductal adenocarcinoma: is tumor budding the missing link?. Front Oncol.

[14] Karamitopoulou E (2012). Tumor budding cells, cancer stem cells and epithelial-mesenchymal transition-type cells in pancreatic cancer. Front Oncol.

[15] NCCN clinical practice guidelines in Oncology (NCCN Guidelines®). http://www.nccn.org/professionals/physician_gls/f_guidelines.asp#.

[16] Peparini N, Chirletti P (2013). Mesopancreas: a boundless structure, namely R1 risk in pancreaticoduodenectomy for pancreatic head carcinoma. Eur J Surg Oncol.

[17] Gaedcke J, Gunawan B, Grade M, Szoke R, Liersch T, Becker H, Ghadimi BM (2010). The mesopancreas is the primary site for R1 resection in pancreatic head cancer: relevance for clinical trials. Langenbecks Arch Surg.

[18] Adham M, Singhirunnusorn J (2012). Surgical technique and results of total mesopancreas excision (TMpE) in pancreatic tumors. Eur J Surg Oncol.

[19] Gockel I, Domeyer M, Wolloscheck T, Konerding MA, Junginger T (2007). Resection of the mesopancreas (RMP): a new surgical classification of a known anatomical space. World J Surg Oncol.

[20] Aimoto T, Mizutani S, Kawano Y, Matsushita A, Yamashita N, Suzuki H, Uchida E (2013). Left posterior approach pancreaticoduodenectomy with total mesopancreas excision and circumferential lymphadenectomy around the superior mesenteric artery for pancreatic head carcinoma. Journal of Nippon Medical School.

[21] Agrawal MK, Thakur DS, Somashekar U, Chandrakar SK, Sharma D (2010). Mesopancreas: myth or reality?. Jop Journal of the Pancreas.

[22] Yeo TP, Hruban RH, Leach SD, Wilentz RE, Sohn TA, Kern SE, Iacobuzio-Donahue CA, Maitra A, Goggins M, Canto MI, Abrams RA, Laheru D, Jaffee EM (2002). Pancreatic cancer. Current Problems in Cancer.

[23] Nakeeb A, Lillemoe KD, Grosfeld JL (2004). Surgical techniques for pancreatic cancer. Minerva Chirurgica.

[24] Golse N, Lebeau R, Lombardbohas C, Hervieu V, Ponchon T, Adham M (2013). Lymph node involvement beyond peripancreatic region in pancreatic head cancers: when results belie expectations. Pancreas.

[25] Jamieson NB, Foulis AK, Oien KA, Going JJ, Glen P, Dickson EJ, Imrie CW, McKay CJ, Carter R (2010). Positive mobilization margins alone do not influence survival following pancreatico-duodenectomy for pancreatic ductal adenocarcinoma. Ann Surg.

[26] Cho BH, Kimura W, Song CH, Fujimiya M, Murakami G (2009). An investigation of the embryologic development of the fascia used as the basis for pancreaticoduodenal mobilization. J Hepatobiliary Pancreat Surg.

[27] Kitagawa H, Tajima H, Nakagawara H, Hayashi H, Makino I, Takamura H, Ninomiya I, Fushida S, Kayahara M, Ohta T, Ikeda H (2013). The retropancreatic fusion fascia acts as a barrier against infiltration by pancreatic carcinoma. Mol Clin Oncol.

[28] Turrini O, Ewald J, Barbier L, Mokart D, Blache JL, Delpero JR (2013). Should the portal vein be routinely resected during pancreaticoduodenectomy for adenocarcinoma?. Ann Surg.

[29] Murakami Y, Uemura K, Sudo T, Hashimoto Y, Nakashima A, Kondo N, Nakagawa N, Sueda T (2013). Benefit of portal or superior mesenteric vein resection with adjuvant chemotherapy for patients with pancreatic head carcinoma. J Surg Oncol.

[30] Sung-Sik H, Sang-Jae P, K Seong Hoon, C Seong Yeon, Young-Kyu K, K Tae Hyun, Soon-Ae L, Myung WS, L Woo Jin, Eun Kyung H (2012). Clinical significance of portal-superior mesenteric vein resection in pancreatoduodenectomy for pancreatic head cancer. Pancreas.

[31] Verbeke CS (2013). Resection margins in pancreatic cancer. Pathologe.

[32] Verbeke CS, Knapp J, Gladhaug IP (2011). Tumour growth is more dispersed in pancreatic head cancers than in rectal cancer: implications for resection margin assessment. Histopathology.

[33] Al-Sukhni E, Attwood K, Gabriel E, Nurkin SJ (2016). Predictors of circumferential resection margin involvement in surgically resected rectal cancer: A retrospective review of 23,464 patients in the US National Cancer Database. Int J Surg.

[34] Tzardi M (2007). Role of total mesorectal excision and of circumferential resection margin in local recurrence and survival of patients with rectal carcinoma. Dig Dis.

[35] Nguyen DX, Bos PD, Massague J (2009). Metastasis: from dissemination to organ-specific colonization. Nat Rev Cancer.

[36] Heidi L (2011). Cancer theory faces doubts. Nature.

[37] Compton CC, Byrd DR, Garciaaguilar J, Kurtzman SH, Olawaiye A (2012). AJCC cancer staging atlas : a companion to the seventh editions of the AJCC cancer staging manual and handbook. Springer.

[38] The Royal College of Pathologists (2002). Standards and minimum datasets for reporting cancers. Minimum dataset for the histopathological reporting of pancreatic, ampulla of Vater and bile duct carcinoma.

[39] Peparini N, Chirletti P (2013). The impact of epithelial-mesenchymal transition on R1 status of the mesopancreatic resection margin after pancreaticoduodenectomy for pancreatic carcinoma: a research proposal topic. Eur J Cancer.

[40] Thiery JP (2002). Epithelial-mesenchymal transitions in tumour progression. Nat Rev Cancer.

[41] Galvan JA, Zlobec I, Wartenberg M, Lugli A, Gloor B, Perren A, Karamitopoulou E (2015). Expression of E-cadherin repressors SNAIL, ZEB1 and ZEB2 by tumour and stromal cells influences tumour-budding phenotype and suggests heterogeneity of stromal cells in pancreatic cancer. Br J Cancer.

[42] DD Von Hoff, Ervin T, Arena FP, Chiorean EG, Infante J, Moore M, Seay T, Tjulandin SA, Ma WW, Saleh MN, Harris M, Reni M, Dowden S (2013). Increased Survival in Pancreatic Cancer with nab-Paclitaxel plus Gemcitabine. New England Journal of Medicine.

[43] Conroy T, Desseigne F, Ychou M, Bouché O, Guimbaud R, Bécouarn Y, Adenis A, Raoul JL, Gourgou-Bourgade S (2011). De lFC. FOLFIRINOX versus gemcitabine for metastatic pancreatic cancer. New England Journal of Medicine.

[44] Tang K, Lu W, Qin W, Wu Y (2016). Neoadjuvant therapy for patients with borderline resectable pancreatic cancer: A systematic review and meta-analysis of response and resection percentages. Pancreatology.

[45] Rashid OM, Pimiento JM, Gamenthaler AW, Nguyen P, Ha TT, Hutchinson T, Springett G, Hoffe S, Shridhar R, Hodul PJ, Johnson BL, Illig K, Armstrong PA (2015). Outcomes of a Clinical Pathway for Borderline Resectable Pancreatic Cancer. Ann Surg Oncol.

[46] Masui T, Doi R, Kawaguchi Y, Sato A, Nakano K, Ito T, Anazawa T, Takaori K, Uemoto S (2016). Concurrent gemcitabine+S-1 neoadjuvant chemotherapy contributes to the improved survival of patients with small borderline-resectable pancreatic cancer tumors. Surg Today.

